# Transcription factor NOR and CNR synergistically regulate tomato fruit ripening and carotenoid biosynthesis

**DOI:** 10.1186/s43897-024-00103-5

**Published:** 2024-07-08

**Authors:** Mengting Liu, Jing Zeng, Ting Li, Ying Li, Yueming Jiang, Xuewu Duan, Guoxiang Jiang

**Affiliations:** 1 State Key Laboratory of Plant Diversity and Specialty Crops & Guangdong Provincial Key Laboratory of Applied Botany, South China, Botanical Garden , Chinese Academy of Sciences, Guangzhou, 510650 China; 2 South China National Botanical Garden, Guangzhou, 510650 China; 3 University of Chinese Academy of Sciences, Beijing, 100049 China

An expanding body of evidence has shown that NAC (NAM/ATAF1/2/CUC2) TF family plays crucial roles in regulating fruit ripening in both climacteric and non-climacteric fruits. In tomato, the NOR (NAC NON-RIPENING) transcription factor has been identified as a master regulator of fruit ripening by activating the expression of genes involved in ethylene synthesis, cell wall degradation, carotenoid biosynthesis, and the flavor formation (Gao et al. 2020, 2022). Additionally, NOR also controls fruit ripening via a positive feedback loop with the essential DNA demethylase DML2 (Gao et al. 2022). Despite its significant role in regulating fruit ripening and quality development, the molecular mechanisms underlying the transcriptional activity of NOR remain largely unknown.

To further explore the regulatory role of NOR in fruit ripening, we utilized NOR as the bait in the yeast two-hybrid (Y2H) system and identified 10 potential NOR-interacting proteins (Table S2), including CNR (COLORLESS NON-RIPENING). Despite significant phenotypic variations between *CNR* spontaneous mutants and knockout mutants, ample genetic evidence has validated *CNR's* essential role in fruit ripening regulation (Manning et al. 2006; Wang et al., 2020; Gao et al. 2019; Lai et al. 2020). Therefore, CNR was chosen for further analysis. Subsequently, the interaction between NOR and CNR was validated using full-length proteins in Y2H (Fig. S1a) and pull-down assays (Fig. S1b). Co-immunoprecipitation (Co-IP) assay demonstrated that CNR binds to NOR in vivo (Fig. 1a), and this interaction was verified to occur in the nucleus through bimolecular fluorescence complementation (BiFC) assay (Fig. 1b, S2a), consistent with the subcellular localization of NOR and CNR (Fig. S2b). These findings indicated that NOR physically interacts with CNR both in vitro and in vivo.

**Fig. 1:**
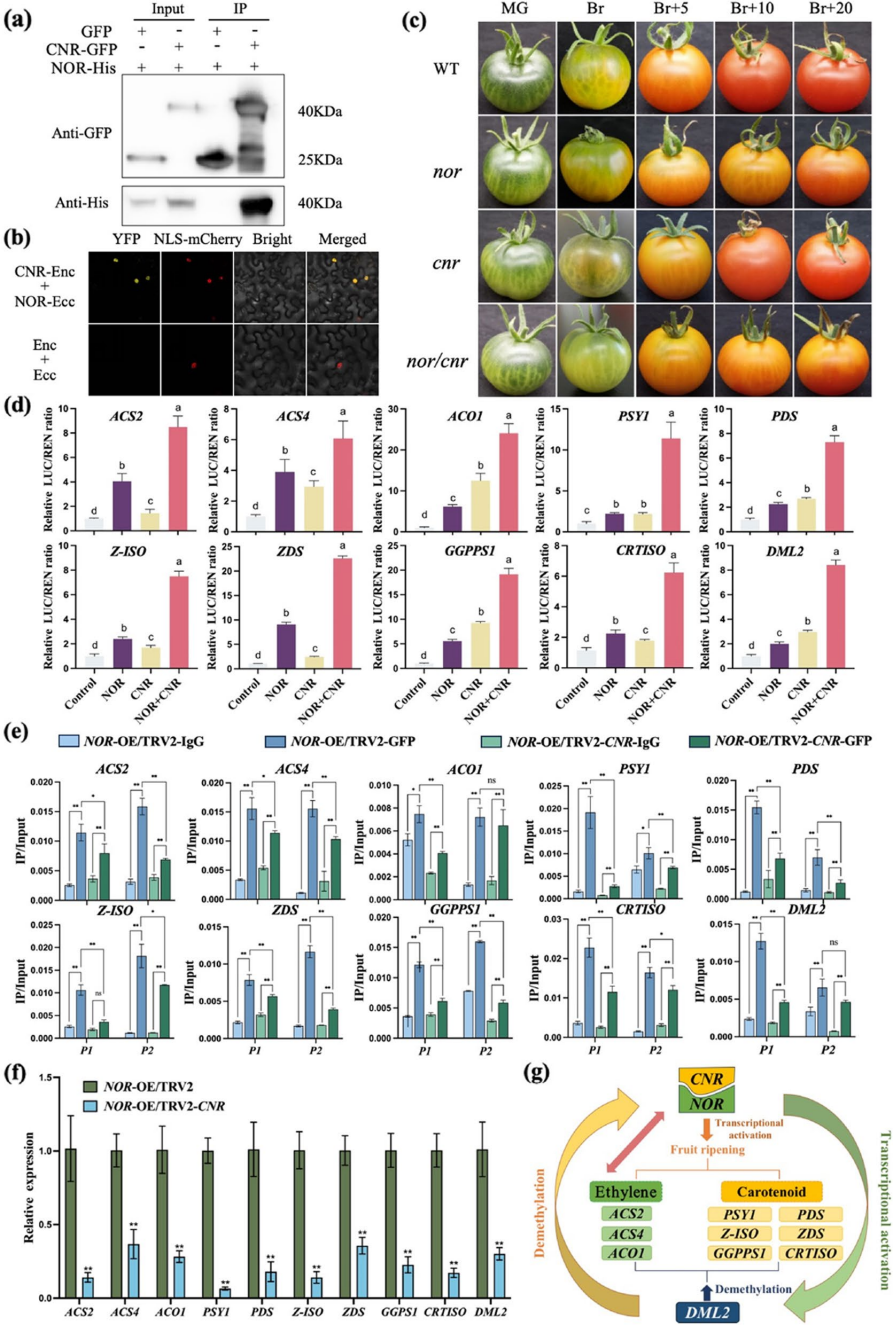
Transcription factor CNR positively regulates tomato fruit ripening through enhancing the transcriptional activity of NOR. **a** CoIP validates the interaction between NOR and CNR. NOR‐His and CNR-GFP were transiently expressed in *N. benthamiana* leaves by Agrobacterium‐mediated infiltration. Total proteins were extracted from leaves and immunoprecipitated with anti‐GFP antibody. Anti‐His antibody was used to detect NOR, and anti‐GFP antibody was used to detect CNR. GFP was used as a negative control. **b** BiFC assays showing the physical interaction between NOR and CNR. NLS-mCherry was used as a nuclear marker. **c** Fruit phenotypes of the WT, *nor*, *cnr*, and *nor*/*cnr* mutants at MG (mature green), Br (break), Br + 5 (break + 5 days), Br + 10 (break + 10 days) and Br + 20 (break + 20 days) stages. **d** Dual-luciferase assays showing the effect of CNR and NOR on the transcriptional activity of potential CNR and NOR co-regulate genes. CNR and NOR were driven by 35S promoter as an effector. The potential CNR and NOR co-regulate genes promoters were used to drive LUC as a reporter. Relative LUC activity as shown on the right. Data are the mean ± SD of six biological replicates. Different letters above the bars indicate statistically significant differences (Student’s t test; *P* < 0.05). **e** ChIP-qPCR assays showing the direct binding of CNR and NOR to the promoters of the potential *CNR* and *NOR* co-target genes. An anti-GFP antibody was used for immunoprecipitation, with IgG serving as the negative control. *P1* and *P2* indicate the amplified regions by ChIP-qPCR. Data are presented as the mean ± SD of three biological replicates. Asterisks above the bars indicate statistically significant differences (Student’s *t* test; *P < 0.05, **P < 0.01. NS no significance). **f** RT-qPCR analysis of potential *CNR* and *NOR* co-regulate genes in *NOR-OE* and *NOR-OE/*TRV2-*CNR* fruits. Data are presented as the mean ± SD of three biological replicates. Asterisks above the bars indicate statistically significant differences (Student’s *t* test; *P < 0.05, **P < 0.01).**g** The proposed model of NOR and CNR in modulating tomato fruit ripening

To assess the collaborative function of these two transcription factors, *nor*, *cnr* and *nor/cnr* mutants were created in the Ailsa Craig (AC) background using CRISPR/Cas9 gene editing technology. As shown in Fig. 1c, the ripening characteristics of *nor/cnr* double mutants were more severely delayed compared to those of *nor* and *cnr* single mutants (Fig. S3). Consistently, fruits from *nor/cnr* double mutants exhibited lower carotenoid levels (Fig. S4a) and higher chlorophyll levels (Fig. S4b) than the *nor* or *cnr* single mutants, implicating that NOR and CNR collaborate to regulate tomato fruit ripening and carotenoid biosynthesis.

To investigate the candidate target genes co-regulated by NOR and CNR, 10 potential genes with NAC and SPL binding sites were selected based on the observed delayed ripening phenotypes and previous published RNA-seq data (Gao et al. 2019), including genes involved in ethylene synthesis (*ACS2*, *ACS4*, *ACO1*)*,* carotenoid biosynthesis (*PDS*, *PSY1*,* Z-ISO*, *CRTISO, ZDS, GGPPS1*)*,* and the DEMETER-like DNA demethylase (*DML2*). RT-qPCR analysis showed that these potential target genes exhibited reduced mRNA levels in pericarp tissues of *nor*, *cnr* and *nor/cnr* mutants compared to the wild-type, particularly in the *nor/cnr* double mutants (Fig. S5). These results indicate that CNR might positively regulate the transcription of NOR-targeted genes.

To clarify the regulatory effects of NOR and CNR on ethylene production and carotenoid biosynthesis, genes associated with these processes were identified for further investigation. Electrophoretic mobility shift assay (EMAS) showed that NOR could specifically bind to the NAC binding sites of the ethylene production (*ACS2*, *ACS4*, *ACO1*) and carotenoid biosynthesis genes promoters (*PDS*, *PSY1*, *Z-ISO*, *CRTISO, ZDS*, *GGPPS1*), while CNR could enhance the binding activity of NOR (Fig. S6-S7). Transcription activity assay in *Nicotiana benthamiana* leaves demonstrated that the individual expression of CNR or NOR could activate the transcriptional activity of ethylene biosynthesis and carotenoid synthesis genes. Notably, co-expression of NOR with CNR significantly boosted the transcription of these ripening-related genes (Fig. 1d). Additionally, chromatin immunoprecipitation (ChIP)-qPCR assay confirmed that NOR directly binds to the promoter of these genes in vivo, while transiently silencing of *CNR* in *NOR-OE* genetic background notably reduced the binding activity of NOR to these target gene promoters (Fig. 1e). Furthermore, the decreased transcript levels of ethylene and carotenoid biosynthesis genes correlated with the diminished binding activity of NOR to these gene promoters in the *NOR-OE* lines with silenced *CNR* (Fig. 1f). These results suggest that NOR may form a transcriptional activation complex with CNR to positively regulate the transcription of ethylene and carotenoid biosynthesis genes.

DNA methylation plays a crucial role in fruit ripening and nutritional quality. In tomato, DML2 has been identified as a master regulator of ripening, acting upstream of the major ripening-related transcription factors RIN, CNR and NOR (Liu et al., 2015; Lang et al. 2017). Furthermore, NOR is implicated in the epigenetic control of fruit ripening by activating *DML2* expression (Gao et al. 2022). The current study indicated that *DML2* expression was notably decreased in *nor*, *cnr* and *nor/cnr* deficient mutants compared to the WT fruits, particularly in the *nor/cnr* double mutants (Fig. S5). EMSA (Fig. S6-S7) and ChIP-qPCR (Fig. 1e) results showed that NOR and CNR bind to the *DML2* promoter both in vitro and in vivo. Additionally, dual-luciferase reporter assays demonstrated that NOR alone significantly enhanced *DML2* transcription in tobacco leaves, while co-expression with CNR markedly boosted the transcription activity of NOR (Fig. 1d). Moreover, transient silencing of *CNR* in the *NOR-OE* genetic background substantially lowered *DML2* expression levels (Fig. 1f). Consistently, the binding activity of NOR to the *DML2* promoter decreased (Fig. 1e).These results suggest a positive feedback loop between NOR/CNR and DML2 in tomato, jointly regulating fruit ripening and carotenoid biosynthesis.

NOR and CNR have long been considered as key regulators of fruit ripening based on natural mutations (Giovannoni et al. 2017). However, recent research has shown that knockout *CNR* displays a ripening-arrested phenotype, whereas knockout *NOR* exhibits a partial non-ripening phenotype (Gao et al. 2019, 2020; Wang et al. 2020). In this study, we uncovered that NOR interacts with CNR to enhance ethylene synthesis and tomato fruit ripening. Moreover, NOR/CNR-mediated *DML2* expression feedback to regulate *NOR/CNR* gene expression. Additionally, NOR/CNR directly triggers *DML2* expression, which in turn indirectly regulates the expression of ripening-related genes through DNA demethylation, thus promoting fruit ripening (Fig. 1g). Our results establish an essential molecular interaction between DML2 and NOR/CNR for fruit ripening and quality formation, indicating that fruit ripening is regulated by a quite complex genetic network. These findings will be beneficial for enhancing fruit quality in tomato breeding.

### Supplementary Information

Supplementary Material 1. Supplementary Materials and Methods.

Supplementary Material 2. Supplementary Figures.

Supplementary Material 3. Supplementary Table S1 and Table S2.

## Data Availability

The data that supports the findings of this study are available in the supplementary material of this article.
