# Supplementary material for: Transcription factor NOR and CNR synergistically regulate tomato fruit ripening and carotenoid biosynthesis
Source: Mol Hortic. 2024 Jul 8;4:27. doi: 10.1186/s43897-024-00103-5 (PMC11232299; doi:10.1186/s43897-024-00103-5)
Supplement: Supplementary file 1 — Supplementary Material 1. Supplementary Materials and Methods. [file 43897_2024_103_MOESM1_ESM.docx]

**Transcription factor NOR and CNR synergistically regulate tomato fruit ripening and carotenoid biosynthesis**

Mengting Liu^1, 2, 3^ , Jing Zeng^1, 2, 3^, Ting Li^1, 2, 3^, Ying Li^1, 2, 3^, Yueming Jiang^1, 2, 3^, Xuewu Duan^1, 2, 3^, Guoxiang Jiang^1, 2, 3, *^

^1^ State Key Laboratory of Plant Diversity and Specialty Crops & Guangdong Provincial Key Laboratory of Applied Botany, South China Botanical Garden, Chinese Academy of Sciences, Guangzhou 510650, China

^2^ South China National Botanical Garden, Guangzhou 510650, China

^3^University of Chinese Academy of Sciences, Beijing 100049, China

**Running title**: NOR and CNR synergistically regulate tomato fruit ripening

**^*^Corresponding authors:**

Guoxiang Jiang, Tel: +86 20 87578854; Email: [gxjiang@scbg.ac.cn](mailto:gxjiang@scbg.ac.cn)

**Materials and Methods**

**Plant materials and growth conditions**

Wild-type (WT) tomato (*Solanum lycopersicum* Mill. Cv Ailsa Craig (AC)) and transgenic plants in the AC background were grown in a condition controlled greenhouse (23 °C with a 16 h/8 h (day/night) cycle). The pericarp of harvested fruits at MG (mature green), BR (break), Br+5 (break + 5 days), Br+10 (break + 10 days) and Br+20 (break + 20 days) stages were immediately frozen in liquid nitrogen and stored in -80 ℃ until use.

**Generation of Transgenic Tomato Plants**

The full-length *NOR* coding sequence was amplified and inserted into the pBI121-GFP vector to create the pBI121-NOR-GFP constructs. The constructs were then transformed into *A. tumefaciens* strain GV3101 to produce *NOR* overexpression lines.

To generate the *cnr* mutants, two specific sgRNA target sites were designed and inserted into the pPTG-sgRNA-Cas9-AtU6-1 vector (Wang *et al*., 2018). The resulting vectors were confirmed by sequencing, then introduced into introduced into *Agrobacterium tumefaciens* strain GV3101, and finally transformed into the cotyledon explants of the tomato cultivar AC, following the method described previously (Jiang *et al*., 2020). The *nor* mutant was kindly provided by Dr. Daqi Fu from China Agricultural University). The nor/*cnr* double mutants were produced by crossing single-mutant *nor* plants with *cnr* plants. Kanamycin-resistant transformants were selected and verified by PCR and DNA sequencing in the T0 and T1 generations. The primers used for vector construction are listed in Supplementary Table S1.

**Virus-induced gene silencing (VIGS)**

VIGS of tomato fruits were performed as previously described (Fu *et al.*, 2005). The specific cDNA fragments of *CNR* were amplified and cloned into the pTRV2 vector to generate pTRV2-*CNR* construct*,* which was then introduced into *A. tumefaciens* strain GV3101. To infiltrate tomato plants, equal amounts of *Agrobacterium* bacteria containing pTRV1 and pTRV2-*CNR* were mixed and injected into inflorescence peduncles attached to the fruit, respectively. The *CNR*-silenced tomato fruits in *NOR-*OE backgrounds at break stage were observed, sampled and analyzed. Each infiltration was repeated three times, with six different plants infiltrated each time. Gene-specific primers were listed in Supplementary Table S1.

**Carotenoid and chlorophyll content determination**

The pericarp of harvested fruits at different ripening stages (MG, Br, Br+5, Br+10 and Br+20) was collected for chlorophyll and carotenoid determination. Chlorophyll and carotenoid contents were extracted and measured as previously described (Forth and Pyke. 2006) and are expressed as μg/g FW. Three biological replicates were performed, with each replicate consisting of six fruits.

**RNA isolation and RT-qPCR assay**

Total RNA was isolated from tomato fruit pericarp using the polysaccharide polyphenol RNA extraction kit (Vazyme, China). The first-strand cDNAs were synthesized from 2 μg of total RNA with the PrimeScript™ RT reagent Kit (TaKaRa, Japan) . RT-qPCR was conducted with SYBR^®^ Premix Ex TaqTM ^II^ (DRR420A, Takara) on an ABI7500 Real-Time PCR System (Thermo Fisher Scientific, Waltham, MA, USA) following the manufacturer’s instructions. Relative gene expression was determined using the 2^−ΔΔCt^ method. SlACTIN7 (Solyc03g078400) was utilized as the internal control. The analysis included three independent biological replicates.  The primers used in this study are detailed in Supporting Table S1.

**Subcellular localization analysis**

The coding sequences of NOR and CNR were subcloned into the PEAQ-GFP vector. The fusion constructs and control vector (PEAQ-GFP vector) were transformed into *A. tumefaciens* strain GV3101 and injected into 4-week-old *Nicotiana benthamiana* leaves. The intensity of the green florescent protein (GFP) was checked by florescence microscope (Leica SP8 STED 3X) after incubation at 22 °C for 48 hours.

**Yeast two-hybrid assay**

To identify the interacting proteins of NOR in a tomato fruit cDNA library, the N-terminal conserved coding sequence of NOR was inserted into pGBKT7 vector as the bait. Subsequently, the pGBKT7-NOR recombinant plasmid and pGADT7-cDNA library were co-transformed into yeast strain AH109 by the lithium acetate method and cultured on SD/-Leu-Trp-His-Ade medium for 3 days. Positive clones were then screened, and the interaction targets of NOR were determined through PCR amplification and sequencing analysis.

To validate the interaction between NOR and CNR, the coding regions of CNR and NOR were cloned into the pGADT7 and pGBKT7 vectors, respectively. Co-transformation of the bait and prey constructs into yeast strain AH109 were performed, and interactions were detected by spotting on SD/-Trp-Leu and SD/-Trp-Leu-His-Ade medium with x-α-gal.

**Bimolecular fluorescence complementation (BiFC) assay**

For the BiFC assay, the coding sequences of CNR and NOR were amplified and inserted into the pNC-BiFC-Enc or pNC-BiFC-Ecc vector to create the pNC-BiFC-NOR-Ecc and pNC-BiFC-CNR–Enc constructs. These constructs were then introduced into *Nicotiana benthamiana* leaves following the method described previously (Jiang *et al*., 2023).After incubation at 22 °C for 24-48 hours, the YFP fluorescence was visualized using a florescence microscope (Leica SP8 STED 3X).

**Pull-down assay**

For the in vitro pull-down assay, the purified MBP-NOR was incubated with GST or GST-CNR fusion protein in pull-down buffer (20 mM Tris-HCl (pH 7.4), 200 mM NaCl, 1 mM EDTA, 1 mM PMSF, and 1 mM DTT) at 4 °C for 4 hours with continous rocking. GST beads (40 μL; Smart-Lifeciences, China) were added and then incubated for another 4 hours. After centrifugation and washing with the pull-down buffer for three times, the pulled-down proteins were eluted by boiling and subjected to SDS-PAGE and Western blotting. The immunoblot analysis was performed with the Anti-MBP (CST; 2396S) and anti-GST (abcam; ab111947) antibodies.

**Co-Immunoprecipitation assay**

To conduct Co-IP assays, the full-length coding sequences of *NOR* and *CNR* were inserted into pEAQ-His and pEAQ-GFP vectors, respectively. *Agrobacterium* GV3101 carrying these constructs were co-infiltrated into *Nicotiana benthamiana* leaves as previously described (Jiang *et al*., 2020). Total proteins were extracted from the tobacco leaves using the extraction buffer (50 mM Tris-HCl, 150 mM NaCl, 2 mM MgCl_2_, 5 mM DTT, 20% glycerin, 1% NP-40, 1 × protease inhibitors). After centrifugation, the supernatant was incubated with 30 μl of GFP-Trap Magnetic Agarose (ChromoTek, China) at 4 °C for 4 hours. The beads were then washed three times with wash buffer (50 mM Tris-HCl, 150 mM NaCl, 2 mM MgCl_2_, 5 mM DTT, 10% glycerin). Subsequently, the bound proteins were denatured by boiling the GFP-Trap Magnetic Agarose in 5 × SDS loading buffer (Cwbio, China), followed by SDS-PAGE analysis. The immunoblot analysis was performed with anti-GFP (abcam; ab290) and anti-His (abcam; ab9180) antibodies.

**Electrophoretic mobility shift assay**

For the EMSA assay, the coding sequences of NOR and CNR were cloned into pMAL-MBP or pMAL-c4x vector, respectively. The recombinant MBP-NOR and GST-CNR fusion proteins were expressed in *Escherichia coli* BL21 (DE3) and affinity purified according to the manufacturer’s protocol. Oligonucleotide probes of the potential *CNR* and *NOR* co-target genes were labeled using the Pierce™ DNA 3' End Biotinylation Kit (Thermo Fisher Scientific) according to the manufacturer’s instructions. Binding and competition reactions were conducted using the Chemiluminescent EMSA Kit (Beyotime, China) following the manufacturer’s instructions. The primers used in this study are described in Supporting Table S1.

**Dual-luciferase reporting assay**

The coding sequences of *NOR and CNR* were amplified and inserted to PEAQ vector to construct the effectors. The 2 kb promoter regions of the potential *CNR* and *NOR* co-target genes were inserted into pGreenII 0800-LUC as reporter plasmids. Then, the effector and reporter plasmids were co-transformed into tobacco leaves using *A. tumefaciens* strain GV3101. After 2 days of co-transformation, the firefly LUC and REN activities were measured using the Dual-Luciferase® Reporter Assay kit (Promega) according to the manufacturer’s instructions. The relative transcriptional activity was indicated by the LUC/REN ratios. The primers used in this study are described in Supporting Table S1.

**Chromatin immunoprecipitation (ChIP)-qPCR Assays**

ChIP assays were conducted following the protocol as previously described (Jiang *et al*., 2023). Tomato fruits from *NOR-OE* and *NOR-OE/TRV2-CNR* transgenic lines at the break stage were harvested and immediately cross-linked with 1% (v/v) formaldehyde. Subsequently, the chromatin from fruit pericarp was extracted and sonicated using a Sonic Dismembrator (Fisher Scientific) to produce fragments of 300-500 bp. The chromatin was then immunoprecipitated with an anti-GFP (Abcam, UK) antibody and anti-immunoglobulin G (Abcam, UK) antibody for 12 hours at 4°C with rotation. The relative enrichment of immunoprecipitated chromatin was assessed by RT-qPCR using the primers provided in Supporting Table S1 and calculated relative to the input. RT-qPCR primers were designed to ﬂank the NOR-binding sites within the promoter of potential co-target genes. The *ACTIN* gene was used as a negative control. Three independent biological replicates were performed for each sample.

**Statistical analysis**

Differences among different treatments were compared using either Student’s t-test or ANOVA followed by Duncan's test in SPSS version 7.5 (SPSS, Inc., Chicago, IL, USA).

**Reference**

Forth D, Pyke KA. The suffulta mutation in tomato reveals a novel method of plastid replication during fruit ripening. *J Exp Bot*. 2006; 57: 1971-1979.

Fu DQ, Zhu BZ, Zhu HL, Jiang WB, Luo YB. Virus-induced gene silencing intomato fruit. *Plant J.* 2005; 43: 299-308.

Jiang GX, Li ZW, Ding XC, Zhou YJ, Lai HM, Jiang YM, Duan XW. WUSCHEL-related homeobox transcription factor SlWOX13 regulates tomato fruit ripening.*Plant Physiol*. 2023; kiad623.

Jiang GX, Zeng J, Li ZW, Song YB, Yan HL, He JX, Jiang YM, Duan XW. Redox regulation of the NOR transcription factor is involved in the regulation of fruit ripening in tomato. *Plant Physiol*. 2020; 183: 671-685.

Wang ZP, Wang SB, Li DW, Zhang Q, Li L, Zhong CH, Liu YF, Huang HW. Optimized paired-sgRNA/Cas9 cloning and expression cassette triggers high-efficiency multiplex genome editing in kiwifruit. *Plant Biotechnol J.*2018; 16: 1424-1433.
