# Supplementary material for: Transcription factor NOR and CNR synergistically regulate tomato fruit ripening and carotenoid biosynthesis
Source: Mol Hortic. 2024 Jul 8;4:27. doi: 10.1186/s43897-024-00103-5 (PMC11232299; doi:10.1186/s43897-024-00103-5)
Supplement: Supplementary file 2 — Supplementary Material 2. Supplementary Figures. [file 43897_2024_103_MOESM2_ESM.docx]

**Transcription factor NOR and CNR synergistically regulate tomato fruit ripening and carotenoid biosynthesis**

Mengting Liu^1, 2, 3^ , Jing Zeng^1, 2, 3^, Ting Li^1, 2, 3^, Ying Li^1, 2, 3^, Yueming Jiang^1, 2, 3^, Xuewu Duan^1, 2, 3^, Guoxiang Jiang^1, 2, 3, *^

^1^ State Key Laboratory of Plant Diversity and Specialty Crops & Guangdong Provincial Key Laboratory of Applied Botany, South China Botanical Garden, Chinese Academy of Sciences, Guangzhou 510650, China

^2^ South China National Botanical Garden, Guangzhou 510650, China

^3^University of Chinese Academy of Sciences, Beijing 100049, China

**Running title**: NOR and CNR synergistically regulate tomato fruit ripening

**^*^Corresponding authors:**

Guoxiang Jiang, Tel: +86 20 87578854; Email: [gxjiang@scbg.ac.cn](mailto:gxjiang@scbg.ac.cn)


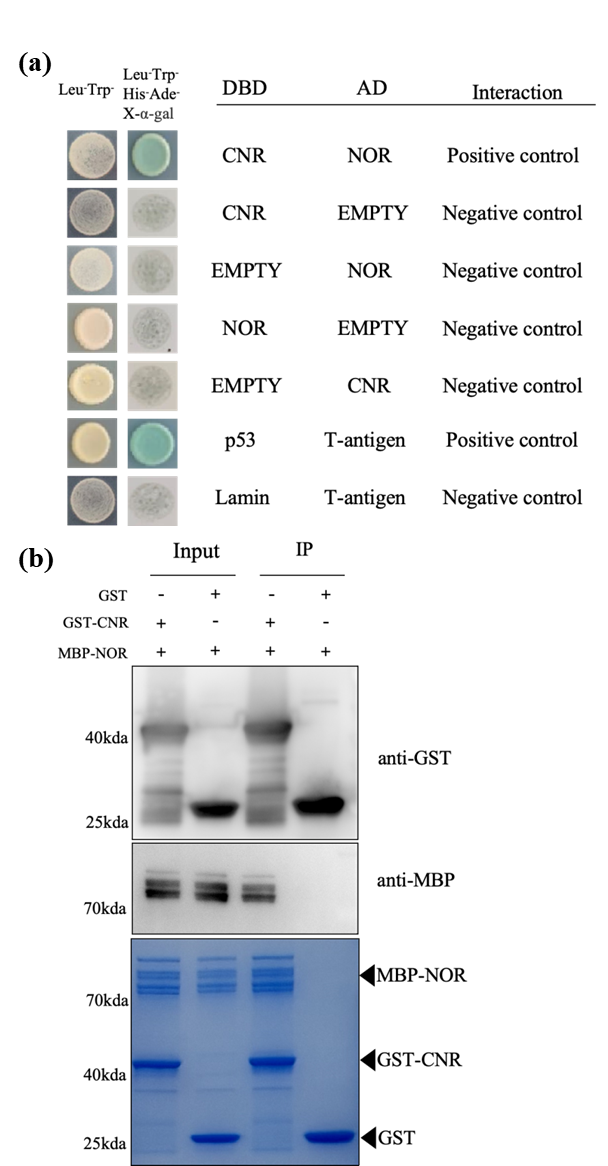


**Fig. S1** (a) Y2H assay revealing the interaction between NOR and CNR. Transformed yeast cells were grown on SD/-Trp/-Leu and SD/-Trp/-Leu/-His/-Ade medium. (b) Pull down assay showing the physical interaction between NOR and CNR. GST-tagged CNR fusion protein (GST-CNR) or GST alone were incubated with MBP-tagged NOR fusion protein (MBP-NOR) in Sepharose 4B beads. MBP-NOR was pulled down by beads containing GST-CNR but not GST alone.


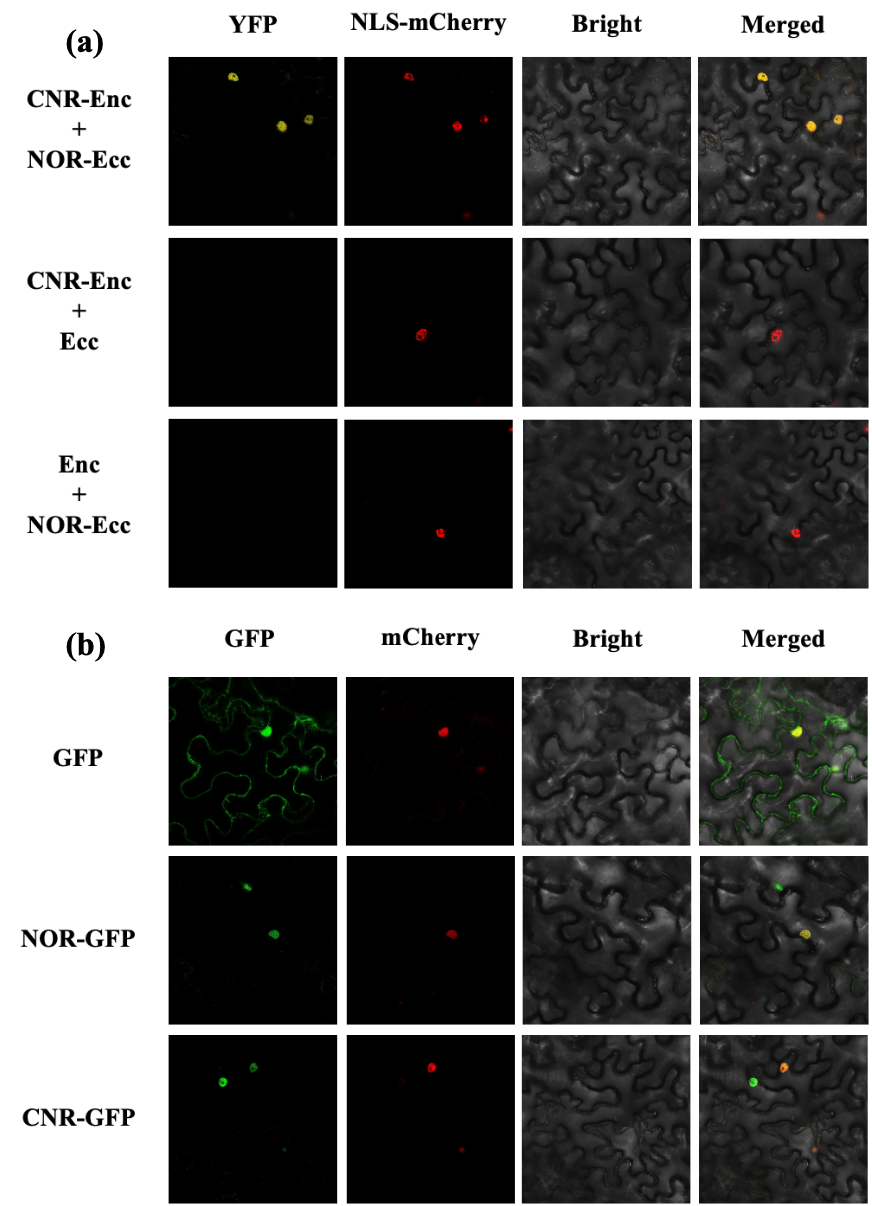


**Fig. S2** (a) Bimolecular fluorescence complementation analysis of NOR interaction with CNR in *N. benthamiana* leaf epidermal cells. NLS-mCherry was used as a nuclear marker. (b) Subcellular localization of CNR and NOR in *N. benthamiana* leaf epidermal cells.


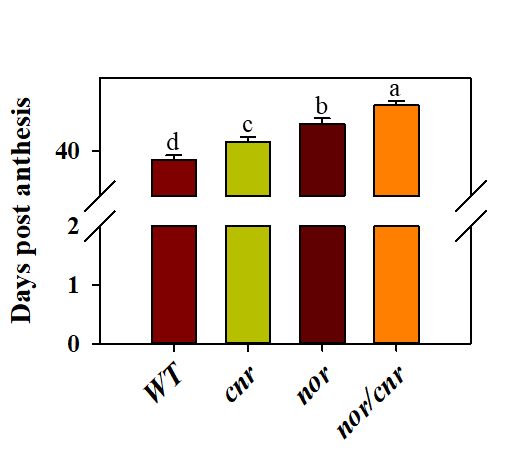


**Fig. S3** The onset of tomato fruit ripening (time from anthesis to break stage) in the *cnr, nor*, and *nor/cnr* mutant compared with WT fruits. Data are the mean ± SD (n=20). Different letters above the bars indicate statistically signiﬁcant differences between the samples (Student’s *t* test; *P* < 0.05).

**
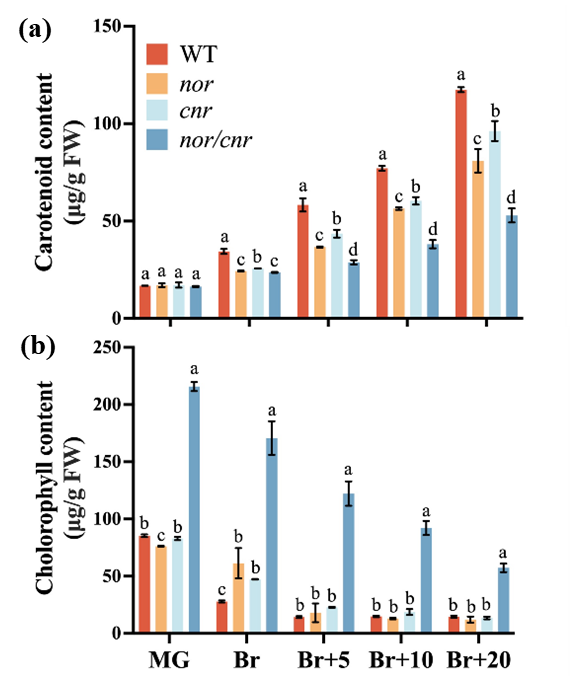
**

**Fig. S4** Carotenoid (a) and Chlorophyll (b) content in the fruits of WT, *nor*, *cnr*, and *nor*/*cnr* mutants. Data are the mean ± SD of six biological replicates. Different letters above the bars indicate statistically signiﬁcant differences (Student’s t test; P < 0.05).


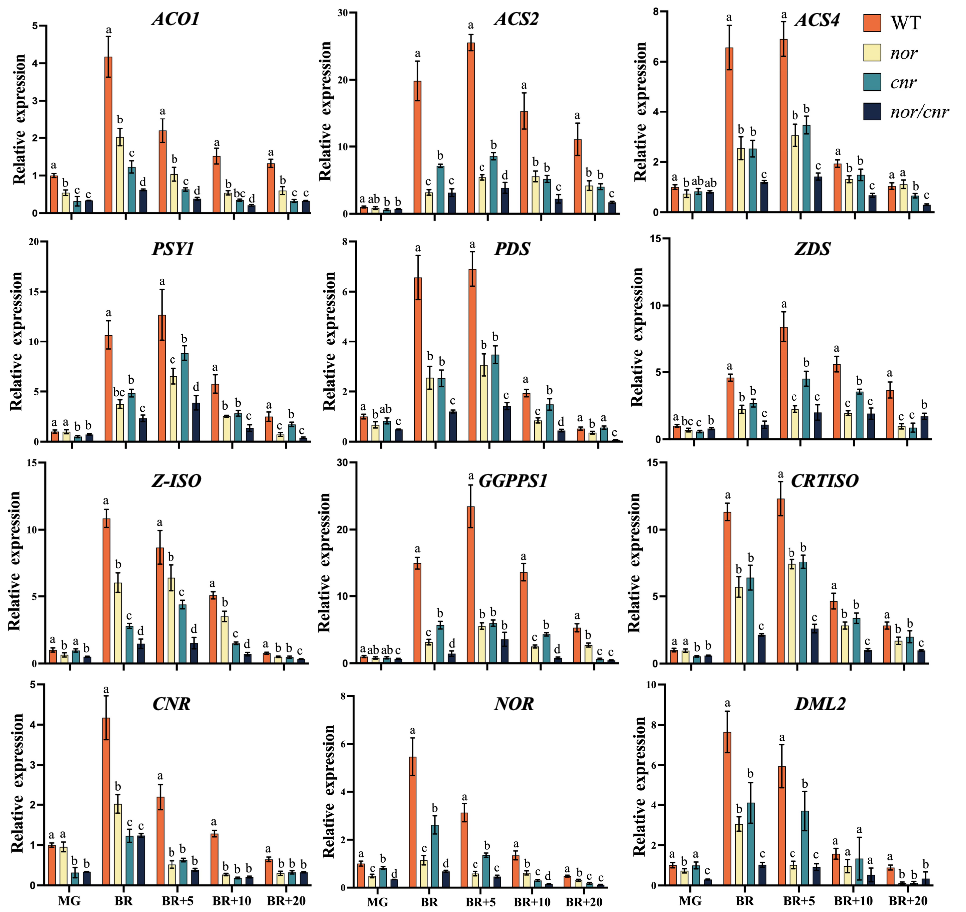


**Fig. S5** Expression of ripening-related genes in the fruits of WT, *nor*, *cnr*, and *nor*/*cnr* mutants. Data are presented as the mean ± SD of three biological replicates. Different letters above the bars indicate statistically signiﬁcant differences (Student’s t test; P < 0.05).


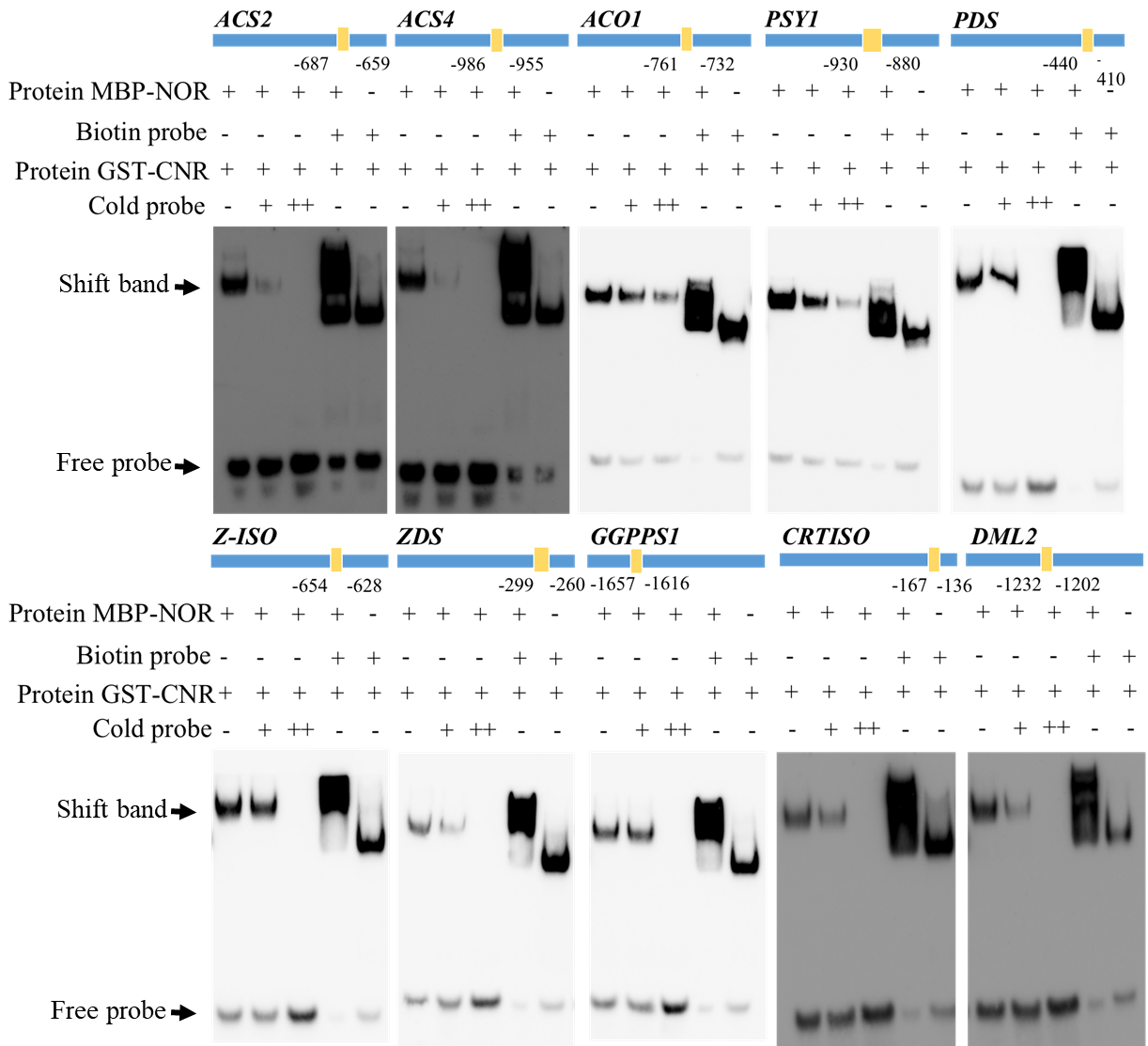


**Fig. S6** EMSA showing that CNR and NOR bound to the biotin-labeled probe present in the promoters of the potential CNR and NOR co-regulate genes. The symbols + or – represent presence or absence, respectively, ++ indicates increasing amounts. The shift band indicating the direct binding of CNR, NOR and CNR/NOR complex to the promoters of the potential CNR and NOR co-regulate genes.

**
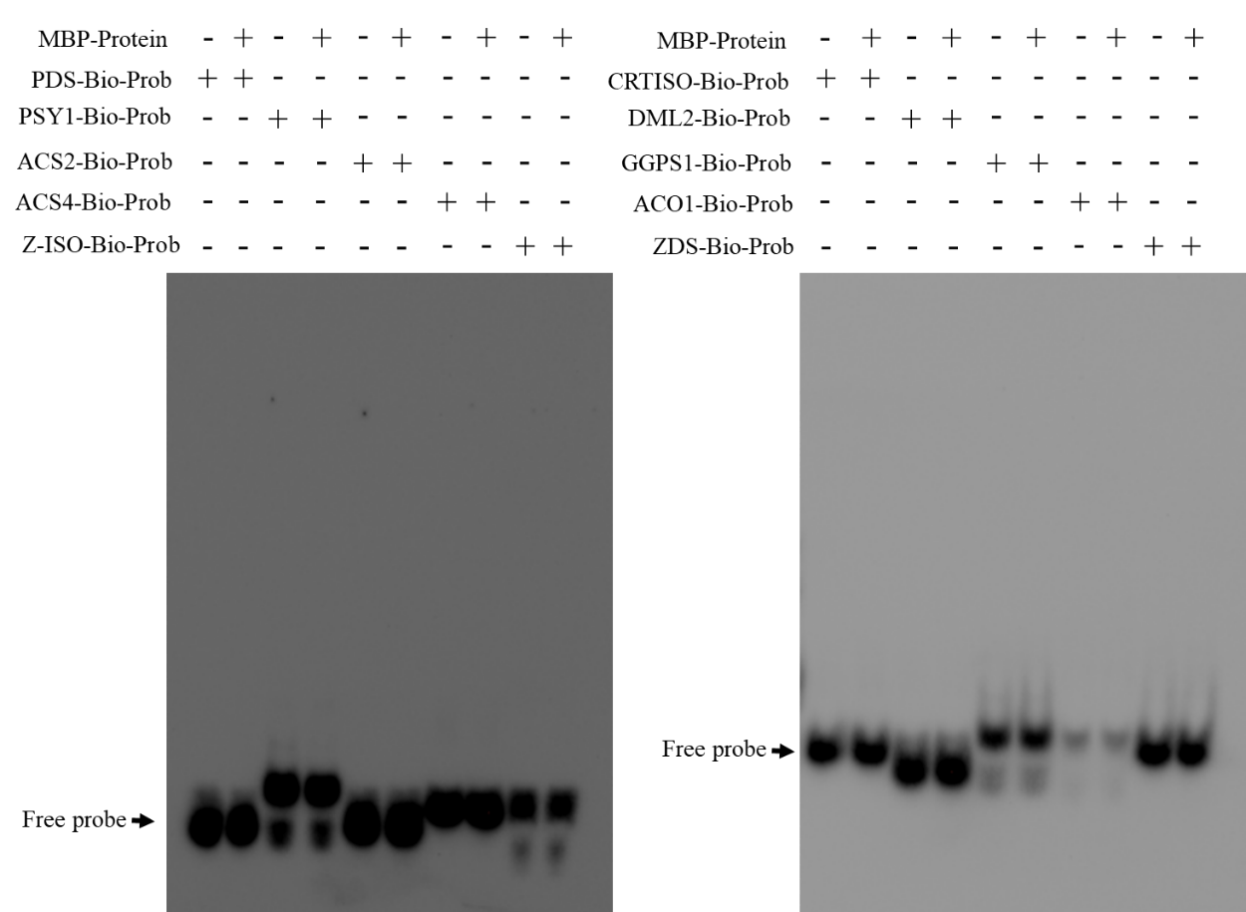
**

**Fig. S7** The negative control of EMSA assay. The symbols + or – represent presence or absence, respectively. The free probe band indicating no direct binding of MBP protein to the promoters of the potential CNR and NOR co-regulate genes.

**
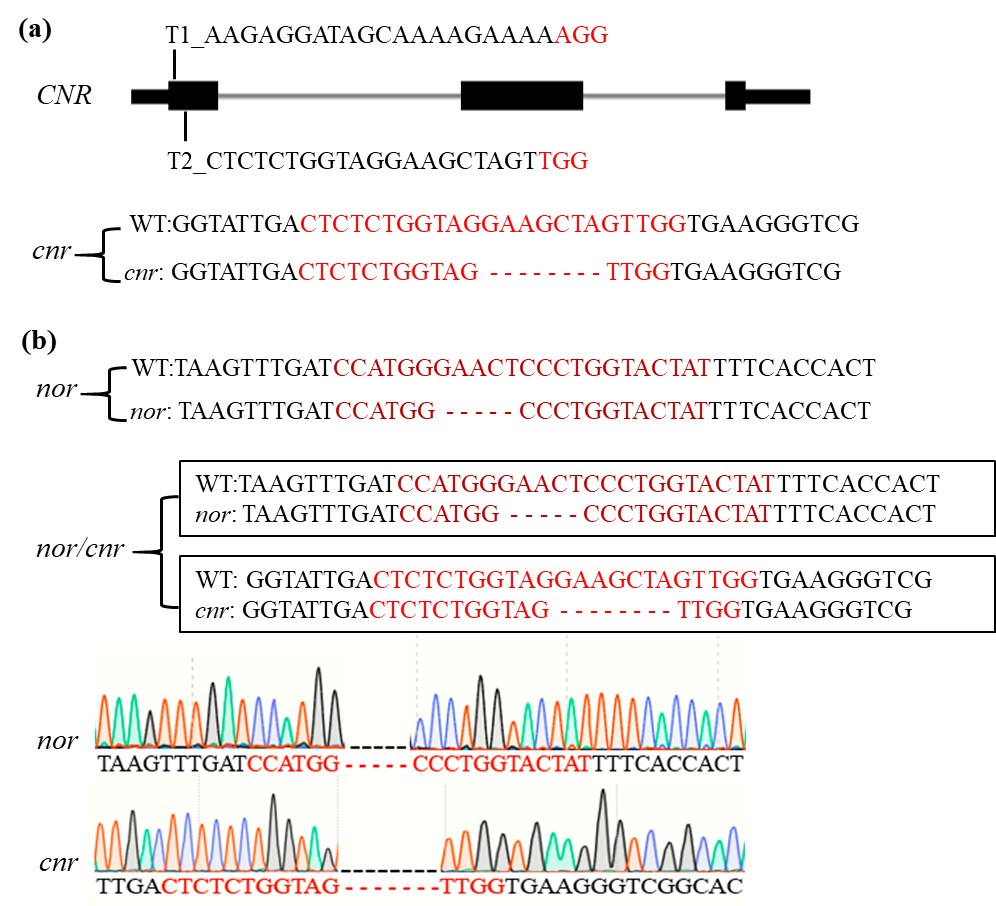
**

**Fig. S8** (a) Generation of *CNR* knockout lines by CRISPR‐Cas9‐mediatedgenome editing in the Ailsa Craig (AC) background.(b) Sanger sequencing of the CRISPR edited sites in *nor* and *nor/cnr* mutants.

**
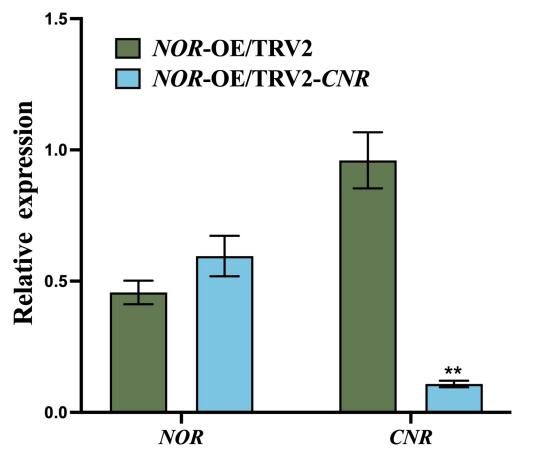
**

**Fig. S9** RT-qPCR analysis of the expression of *NOR* and *CNR* in *NOR-OE* and *NOR-OE/TRV2-CNR* fruits. Data are presented as the mean ± SD of three biological replicates. [Asterisk](javascript:;)s above the bars indicate statistically signiﬁcant differences between the samples (Student’s *t* test; *P* < 0.01).


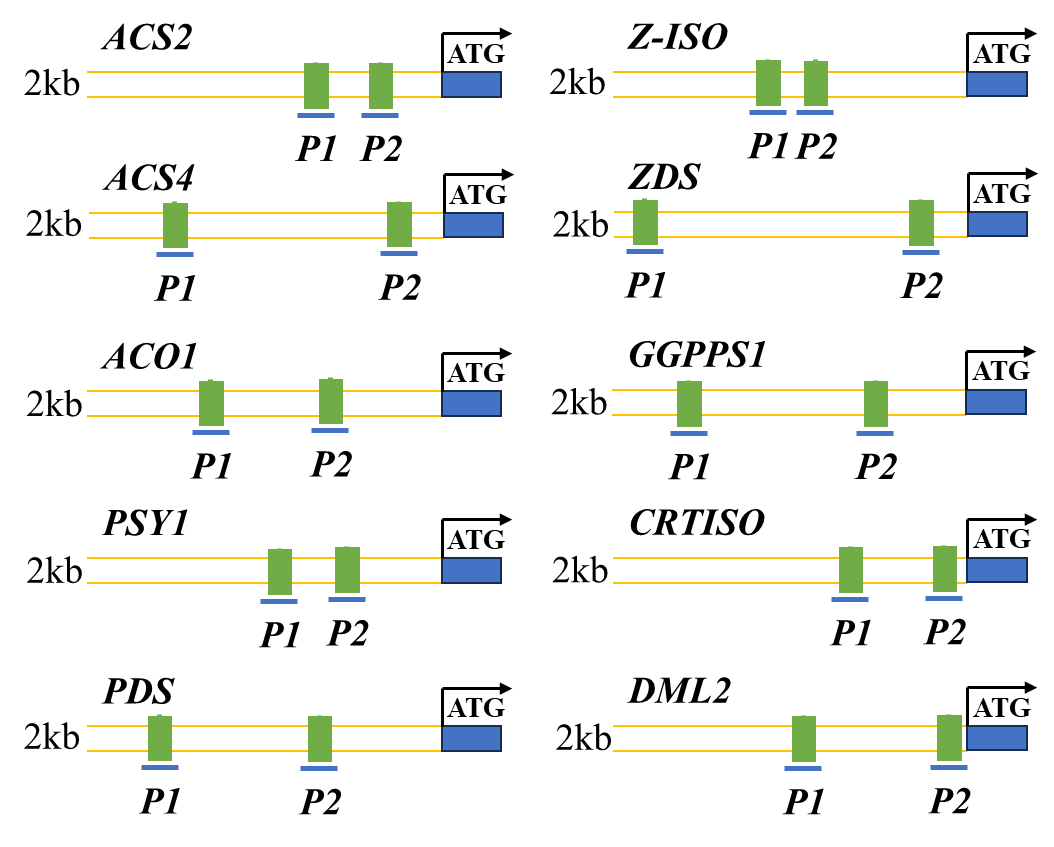


**Fig. S10** Schematic diagram of promoters and primer positions used in ChIP-qPCR assays. *P1* and *P2* indicate the amplified regions by ChIP-qPCR.

**
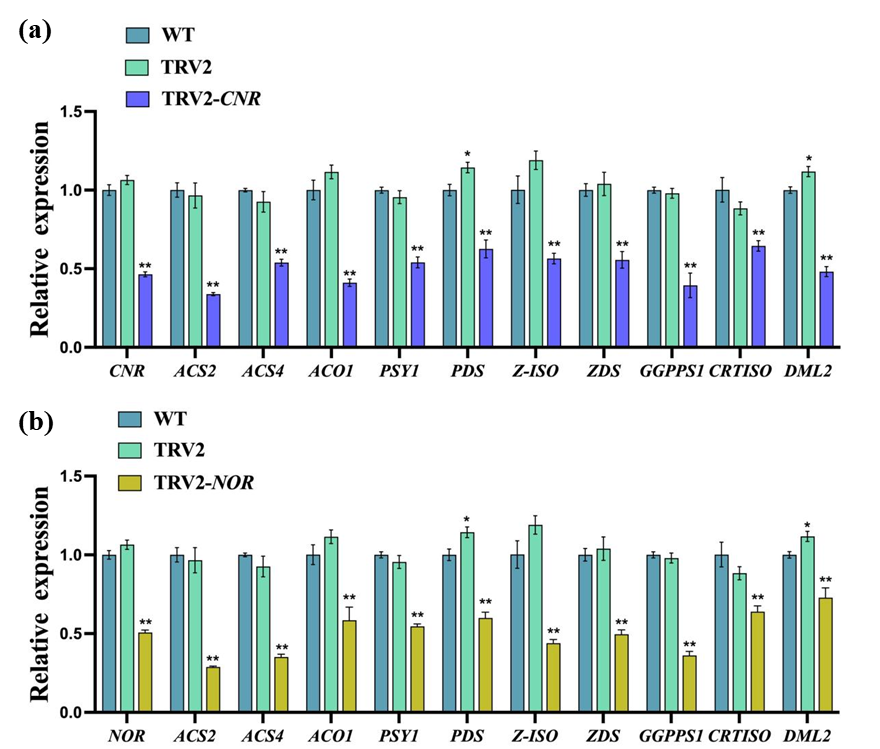
**

**Fig. S11** RT-qPCR analysis of the expression of the CNR and NOR co-regulate genes. (a) Expression of ripening-related genes in the fruits of WT, WT/TRV2, WT/TRV2-*CNR*. (b) Expression of ripening-related genes in the fruits of WT, WT/TRV2, WT/TRV2-*NOR*. Data are presented as the mean ± SD of three biological replicates. [Asterisk](javascript:;)s above the bars indicate statistically signiﬁcant differences (Student’s *t* test; **P* < 0.05, ***P* < 0.01).
